# Artificial intelligence assimilation shapes sustainable performance through dynamic capabilities

**DOI:** 10.1038/s41598-026-46291-6

**Published:** 2026-04-08

**Authors:** Mingyang Zhang, Wenwen Dong

**Affiliations:** 1 School of Public Administration, Sichuan University, Chengdu, China; 2 School of Public Affairs, Xiamen University, Xiamen, China

**Keywords:** AI assimilation, Sustainable performance, Organizational agility, Organizational resilience, Green innovation, Business and management, Business and management, Complex networks, Complex networks, Information systems and information technology, Science, technology and society

## Abstract

Beyond the hype of disruptive technologies, achieving sustainability requires assimilating artificial intelligence (AI) into organizational dynamic capabilities. Yet conflicting evidence on the sustainability outcomes of AI assimilation (AIA) motivates this study to unveil its underlying capability logic. Grounded in the Natural Resource-Based View and Dynamic Capabilities Theory, this study employs meta-analytic structural equation modeling (MASEM) to systematically investigate the intrinsic mechanisms through which AIA affects firms’ sustainable performance (SP). The findings reveal several key insights. First, AIA directly enhances SP and exerts indirect effects through the multiple mediating pathways of organizational agility (OA), green innovation (GI), and organizational resilience (OR). Second, GI serves as a core mediating pathway, playing a pivotal role in connecting AIA with sustainable development goals. Third, OA generates performance gains through short-cycle resource reconfiguration, whereas OR provides stable support in uncertain environments; these two capabilities exhibit complementary characteristics across different time scales. Finally, the research elucidates the complete pathway wherein AIA influences SP through the serial mediation of OA, GI, and OR. This reveals the underlying logic of how AI drives sustainable development through the evolution of dynamic capabilities. By integrating findings across existing studies, this research helps to reconcile discrepancies in prior conclusions. It provides a novel theoretical explanation for understanding the complex mechanisms of AI-enabled corporate sustainable development and also offers practical implications for businesses advancing digital and intelligent transformation.

## Introduction

Artificial intelligence (AI) is reshaping the competitive landscape by enhancing operational efficiency, deepening customer insight, improving resource allocation, and strengthening decision quality, thereby accelerating organizational transformation^1–4^. Yet firms experience markedly uneven returns from AI initiatives, suggesting that technological capability alone rarely translates into replicable and scalable performance advantages. Realizing AI’s business value depends more critically on aligned process redesign, robust data governance, and cross-functional collaboration that enable AI to be routinely leveraged in operations and managerial decision-making^4^. Accordingly, attention should move beyond adoption toward the organizational process through which AI becomes continuously embedded and institutionalized in core processes and decision routines, ultimately constituting a durable capability. This deep integration and routinization are commonly conceptualized as artificial intelligence assimilation (AIA)^5,6^.

At the same time, sustainable development has become central to long-term competitiveness, requiring firms to deliver synergistic outcomes across economic, environmental, and social dimensions^7^. In principle, AI aligns with sustainability imperatives by enabling data-driven decision-making, improving resource efficiency, and strengthening prediction and risk management, with potential spillovers to green innovation and environmental performance^8,9^. However, whether and how AIA can systematically convert technological potential into sustainable performance (SP) remains theoretically unsettled and empirically inconsistent. A systematic assessment of the pathways linking AIA to SP is therefore warranted. Such an effort can unpack the “black box” that connects technological assimilation to sustainable value creation and can inform managerial choices under high investment and high uncertainty, including the pacing of AI investments, the allocation of complementary organizational resources, and the design of governance arrangements.

Prior research has not reached consensus on the AIA–SP relationship. One stream suggests that embedding AI into key processes and integrating AI-enabled functions across departments enhances information processing and decision quality, thereby improving comprehensive performance^2^. Related arguments emphasize that AI’s business value is driven not by deployment alone but by the depth of assimilation and process-level reconfiguration^4^. Evidence further indicates that assimilation practices oriented toward collaboration and responsiveness can strengthen operational outcomes relevant to sustainability^10^. In contrast, another stream highlights substantial sunk costs, governance complexity, and organizational change frictions associated with AI implementation, leading to highly heterogeneous returns across industries and firms and, in some settings, adverse net effects^11^. In addition, the diffusion of automation may generate asymmetric labor-market and welfare consequences, potentially constraining the social dimension of performance^12^. AI project failures are also frequently attributed to organizational and governance constraints, indicating that technological sophistication does not guarantee performance gains^13^, and a persistent management paradox emerges between automation and augmentation objectives^14^. Taken together, cumulative evidence has not yet converged on either the direction or the robustness of the baseline association between AIA and SP.

To reconcile these mixed findings, scholars have proposed multiple pathways operating at different levels. First, an efficiency and allocation pathway posits that AIA improves SP by enhancing productivity and resource allocation efficiency^15^, with supply chain research further suggesting that AI-enabled digital capabilities shape resilience and performance realization^16^. Second, an innovation stimulation pathway conceptualizes AIA as a catalyst for knowledge recombination and exploration, thus facilitating green technological innovation and environmental goal attainment^17^; correspondingly, green innovation may play an important mediating role linking AI capability to sustainable outcomes^18^. Third, a capability-building pathway argues that AIA strengthens a firm’s ability to sense and respond to uncertainty, enhancing organizational agility and resilience^19^. Operations and supply chain research similarly treats agility and resilience as key mechanisms through which digital capabilities translate into performance^20^, and recent work has begun to examine more directly how AIA relates to agility and SP^21^. More broadly, digital transformation studies also connect platform-based capabilities and strategic digitalization to sustainable performance, including evidence from cloud manufacturing platforms and SME digital strategies in emerging markets^22,23^, as well as analyses of multidimensional barriers to digital disruption in clean cloud manufacturing contexts^24^.

Despite these advances, three gaps remain. First, reported AIA–SP effects appear highly contingent on industry conditions, institutional environments, and measurement choices, while AIA has been increasingly discussed, cumulative evidence on effect stability and generalizability remains limited^6^. Second, mediation is often examined via a single mechanism or a small subset of mechanisms, providing limited comparative and testable evidence on the relative importance of parallel and serial pathways, thereby constraining theory refinement and empirical accumulation. Third, existing studies more frequently explain short-term efficiency or responsiveness gains from assimilation, but offer less coherent logic on how such gains become institutionalized as sustainability-oriented capabilities that consistently manifest in triple-bottom-line performance, an important reason why findings remain difficult to generalize across contexts.

Addressing these gaps requires moving beyond a purely technological determinism account and adopting an integrative framework that captures both the value attributes of AI-related resources and the evolutionary development of organizational capabilities. The Natural-Resource-Based View (NRBV) offers a strategic lens for understanding how firms deploy resources to respond to environmental pressures and create multidimensional value^25^. Dynamic Capability Theory (DCT) complements this perspective by explaining how firms transform resources into institutionalized capabilities through sensing, seizing, and reconfiguring in turbulent environments^26^. Integrating NRBV and DCT enables a more complete chain of explanations linking short-term response, value-creating actions, and the formation of long-term adaptive capacity. By integrating NRBV and DCT with a cumulative meta-analytic structural equation modeling (MASEM) design, this study offers a mechanism-centered synthesis of how AIA translates into SP. It clarifies the robustness of the AIA–SP relationship while adjudicating competing mediation pathways and their sequencing across agility, green innovation, and resilience.

Building on this integrated view, this study employs MASEM to synthesize fragmented empirical evidence on AIA and SP. By aggregating effect sizes across studies, this approach enables the estimation and testing of complex multi-path structural models at a higher level of inference, reducing sensitivity to idiosyncratic sample characteristics, measurement error, and contextual biases, while allowing the assessment of both direct effects and serial mediation^23,27,28^. Specifically, this research aims to: (1) establish the statistical robustness of the AIA–SP relationship across contexts by synthesizing cumulative empirical findings; (2) uncover transmission mechanisms by testing the relative importance and potential complementarities among organizational agility, green innovation, and organizational resilience as mediators through the dual lens of NRBV and DCT; and (3) validate an evolutionary capability-development logic in which agility enables responsive action, which supports innovation practices, and ultimately contributes to resilience as a protective capacity, thereby offering a capability-based explanation for sustainable competitive advantage in the era of intelligent transformation.

## Theoretical Framework and Hypotheses

### Theoretical framework

To explore the deep mechanism through which AIA drives SP, it is necessary to construct a theoretical framework that integrates value orientation and process transformation. Focusing solely on the procedural dimension of technology application fails to clarify its foundation in value rationality; meanwhile, merely emphasizing the goal dimension of sustainable development cannot reveal the instrumental rationality path from resources to performance. The complementary nature of the Natural Resource-Based View (NRBV) and the Dynamic Capabilities Theory (DCT) provide an appropriate theoretical foundation for bridging this “value-process” divide.

The NRBV establishes the value norms and strategic content orientation for sustainable development. This theory internalizes ecological constraints as endogenous variables for firms to gain competitive advantage and points out that green capabilities such as pollution prevention and product management constitute the foundation for achieving triple bottom line performance beyond economic profits^7,25^. However, the traditional NRBV is primarily based on static analysis of resource stocks. Faced with the increasing complexity of the global environmental and policy network and the accelerating iteration of digital technologies^9^, this theory struggles to fully reveal how organizations dynamically sustain such green advantages through internal process reconfiguration, which constitutes its explanatory boundary in the digital era. The introduction of DCT addresses this gap in the process mechanism. This theory focuses on the dynamic evolutionary process of updating the capability base through cyclical mechanisms of sensing and reconfiguring^26,29^. Especially in the context of digital transformation, digital resource investments must undergo strategic organizational absorption and digestion to be transformed into inimitable heterogeneous capabilities^22^. Therefore, this study aims to construct an integrative analytical lens of complementary value and process within organizations: the NRBV defines the value direction of resource transformation and capability building, while DCT delineates the dynamic mechanism for realizing this value. Their combination, through the transformation of resource potential into capability kinetics, explains the complete pathway of how AIA, catalyzed by dynamic capabilities, ultimately leads to SP.

Based on the above theoretical integration, this study needs to clarify: Can AI technology resource inputs generate SP? Which organizational capabilities constitute the key mediating mechanisms translating AIA into SP? First, AIA serves as the resource-side origin of this study’s logical progression. Unlike superficial technology adoption, assimilation refers to the deep embedding of AI technology into organizational routines and decision-making systems, endowing it with the valuable and scarce characteristics of a strategic resource. Fosso Wamba et al. (2024) note that such deep embedding is a prerequisite for digital technologies to generate supply chain performance^30^. Zhong and Song (2025) confirm that AI technology, by optimizing resource allocation efficiency, constitutes a core antecedent driving green transformation^18^. Kavre et al. (2025) also emphasize that digital resource inputs must undergo organizational absorption and transformation to evolve into heterogeneous competitive advantages^22^. Integrating the principles of environmental protection and sustainable development into AI information governance mechanisms helps minimize environmental risks and social responsibility costs, ultimately enhancing corporate long-term sustainability^31^. Therefore, AIA is not merely a technological deployment act but a strategic resource investment triggering organizational capability reconfiguration.

Second, DCT emphasizes that firms should possess acute sensing abilities to respond to instantaneous environmental changes^26,29^. The core of sensing capability lies in the rapid capture and immediate response to external signals, making organizational reaction speed and tactical flexibility crucial. AI can significantly enhance a firm’s immediate response speed in complex environments and promote green innovation^32^. AI-driven predictive analytics enhance decision-making by improving its agility and visibility, thus empowering organizations to proactively respond to potential supply chain disruptions^33^. Furthermore, especially during crises, purposeful agile action allows organizations to reconfigure resources and adjust strategies to withstand external shocks, thereby ensuring sustained sustainability^34,35^. Therefore, from the perspective of organizational demands for short-term response speed, organizational agility (OA) serves as a vital link connecting AIA and SP.

Third, sustainable competitive advantage depends on a sustained commitment to heterogeneous resources^25,36^. This advantage also requires self-repair and functional evolution, which are achieved through the reconfiguration dimension of dynamic capabilities^26,29,37,38^. Both theoretical perspectives address the central issue of attaining long-term value via the ongoing renewal of organizational capabilities. To navigate rapidly evolving environments and technological disruptions, organizations must cultivate resilience. This concept denotes the capacity to withstand operational interruptions, sustain core functions, and accomplish progressive renewal^39,40^. In contexts shaped by AI, resilience forms the basis for adaptability and organizational learning. It encompasses not only an organization’s ability to survive and recover from turbulence but also its potential to adapt and flourish under conditions of uncertainty^41^. Organizational resilience (OR) emerges when AI applications are effectively integrated with dynamic organizational capabilities and leadership competencies^42^. Moreover, scholarly work has established a connection between OR and sustainable development, pointing to a synergistic relationship between the two^43,44^. The core abilities that constitute resilience, namely anticipating potential disruptions, responding effectively to crises, and adapting to changing circumstances, enable organizations to maintain stability and ensure operational continuity during unforeseen events^45^. This capability subsequently underpins their long-term sustainable development and competitive advantage. Consequently, OR is a vital capability that allows technological resources to be transformed into SP.

Furthermore, achieving SP requires organizations to accomplish a dual transformation: first, translating sustainable value commitments into verifiable practices, and second, institutionalizing dynamic capability renewal into replicable routines of action^25,38^. Green innovation (GI) serves as the key mechanism that carries this dual theoretical demand. It directs technological potential toward the research and development of green products and processes, providing a necessary pathway for AI technologies to enable sustainable development^46^. GI helps organizations leverage AI to predict green demand, monitor environmental impact, and optimize operational feedback, thereby enhancing environmental performance^47–49^. This process is closely linked to OA and OR. OA accelerates the responsiveness and iteration of GI by improving the efficiency of opportunity identification and resource reconfiguration^50–53^. Meanwhile, the accumulated green knowledge and stabilized processes from GI strengthen the functional continuity and recovery capacity of organizations under impact, forming an important foundation for resilience^32,54,55^. Therefore, green innovation constitutes a core link that connects AIA with SP, effectively synergizes organizational agility and resilience.

Finally, SP constitutes the fundamental orientation for the application of AI technologies and the development of organizational dynamic capabilities. The integration of AI by organizations aims not only to enhance operational efficiency and economic benefits but, more critically, to achieve the synergistic advancement of environmental improvement and social value creation. Organizations should adopt a holistic performance measurement framework that incorporates environmental and social dimensions into the core criteria for evaluating organizational success^7,56^. The NRBV internalizes ecological constraints as prerequisites for strategic formulation, identifying green capabilities such as pollution prevention and product stewardship as key sources for building long-term competitive advantage^25^. The institutional environment continuously reinforces requirements for sustainability disclosure and governance compliance. Consequently, organizations need to rely on verifiable sustainable outcomes to consolidate their legitimacy and transform this legitimacy into advantages in terms of value recognition and access to critical resources^57,58^.

**Fig. 1:**
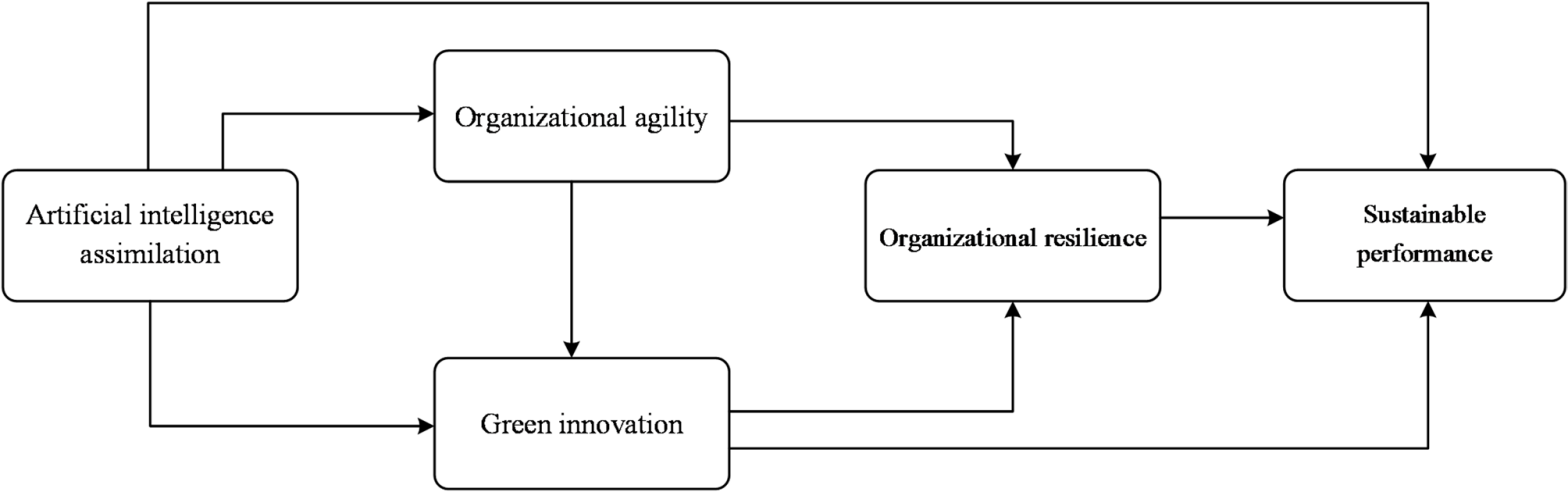
Research framework.

In summary, this study constructs a systematic theoretical analytical framework. The framework takes AIA as the starting point, employs OA and OR to reflect the dual dimensions of dynamic capabilities, utilizes GI as the key practical transformation vehicle, and ultimately positions SP as the comprehensive value endpoint (as shown in Fig. 1). This framework aims to reveal the theoretical pathway from technological resources to sustainable outcomes, providing a theoretical foundation for subsequent research and hypothesis development.

### Hypothesis development

#### AI assimilation, organizational agility, green innovation, and sustainable performance

DCT emphasizes that firms operating in highly uncertain environments require the capacity to sense, seize, and reconfigure in order to sustain competitive advantage^26^. AI assimilation (AIA) embeds AI resources into a firm’s processes and systems, evolving them from a technological deployment into a repeatable organizational capability^13,19^. Organizational agility (OA), which reflects a firm’s ability to rapidly perceive external environmental changes and respond promptly, constitutes a concrete manifestation of dynamic capabilities at the operational level^29^. This process enhances the firm’s responsiveness in areas such as high-frequency sensing, rapid decision-making, and process reconfiguration. Existing research indicates that once AI is embedded into business processes, it can increase the speed at which a firm identifies market signals and its flexibility in responding, thereby enhancing OA^59,6,60^. Mikalef and Gupta^2^ further note that AI capability positively influences the rapid capture and exploitation of market opportunities. The preceding arguments lead to the following hypothesis:

##### H1

AIA has a positive effect on OA.

When firms introduce environmentally oriented green innovation (GI) activities into their products, processes, or management practices, the goal is to achieve a synergistic improvement of both ecological and corporate performance^61^. The NRBV posits that firms can proactively respond to environmental regulations and resource constraints through technological and managerial innovation, thereby carving out a path for green competitive advantage^25,62^. From this perspective, technological capability constitutes a key underpinning for sustainability-oriented strategy. The applications of AI in green innovation include product design optimization, process monitoring, emission control, and enhancement of energy utilization efficiency^8^. By embedding AI into innovation processes, firms can effectively reduce the trial-and-error costs and transformation challenges associated with GI, while improving the coupling efficiency between environmental goals and business practices. Research indicates that AI adoption significantly boosts corporate green patent output and green technology performance^1,63^. Wang et al.^64^ further point out that AI exerts direct, indirect, and spillover effect mechanisms on green innovation. Based on the above analysis, the following hypothesis is proposed:

##### H2

AIA has a positive effect on GI.

The sustainability-oriented technological and managerial practices emphasized by the NRBV contribute to achieving the unification of resource efficiency, risk control, and reputation enhancement, thereby improving financial performance^25,62^. DCT further stresses that firms need to build institutionalized mechanisms within the organization to enable emerging technological resources to deliver sustained value^29^. Sustainable performance (SP) reflects a firm’s comprehensive performance across economic, environmental, and social dimensions, emphasizing the synergistic achievement of multiple objectives^7^. The process of AIA holds the potential to enhance organizational SP across these multiple levels. In the economic dimension, AIA enhances operational efficiency and the precision of resource allocation through process optimization and data-driven decision-making^2^. In the environmental dimension, AI supports pollution monitoring, energy management, and environmental risk early warning, thereby improving environmental performance^8^. In the social dimension, AI promotes process transparency, stakeholder communication, and the capacity for responsibility and compliance, consequently strengthening social performance^65^. Research has found that the deep integration of AI within an organization significantly enhances its overall corporate performance^4^. Benzidia et al.^10^ also indicate that AI has a positive impact on corporate environmental performance. Accordingly, the following hypothesis is proposed.

##### H3

AIA has a positive effect on SP.

#### Organizational agility, organizational resilience, and sustainable performance

According to DCT, an organization’s ability to sustain competitive advantage in a dynamic environment relies on its capacity to sense, seize, and reconfigure resources and processes^26,29^. OA, which reflects the ability to rapidly perceive external changes and adjust strategies and resource allocation, is a concrete manifestation of dynamic capabilities at the operational level^66^. OR, in contrast, focuses on the capacity to maintain functional stability, achieve effective recovery, and facilitate learning when faced with disruptions, corresponding to the reconfiguring dimension of dynamic capabilities^39,67^. While they differ in their temporal focus and functional emphasis, together they constitute a core mechanism for organizational adaptation to complex environments.

OA lays the foundation for the development of OR by enhancing the organization’s response speed and structural flexibility. An agile organization can identify potential risks earlier and accumulate experience in handling non-routine situations during daily operations. This experience gradually solidifies into organizational preparedness for emergencies and mechanisms for coordinated response. When confronting major disruptions, these mechanisms can shorten recovery cycles and enhance the organization’s ability to maintain functionality under stressful conditions. Recent research also indicates that dynamic capabilities often influence performance by enhancing resilience, and the rapid adaptation capacity supported by agility translates into more stable capabilities for withstanding pressure and recovering^68^. Therefore, the following hypothesis is proposed:

##### H4

OA has a positive effect on OR.

OA provides crucial organizational conditions for GI. It enhances the capacity for rapid redeployment of cross-departmental resources and the reintegration of knowledge, enabling firms to swiftly identify changes in regulatory requirements, market preferences, and societal expectations. This allows them to advance green process improvements, redesign energy-saving and consumption-reducing workflows, and develop green products^29,61^. On the other hand, OA can increase decision-making speed^69^ and reduce organizational inertia and path dependency^70,71^, facilitating the institutionalization of digital technologies, data analytics, and management practices as part of routine operations. Within DCT, OA drives the cyclical process of sensing, seizing, and reconfiguring, thereby continuously accelerating the generation of GI outcomes^72^. Accordingly, the following hypothesis is proposed:

##### H5

OA has a positive effect on GI.

OR enables firms to maintain business continuity, preserve relationships with stakeholders, and adhere to long-term oriented green strategies when facing external shocks^73^. The effect of OR on SP can be understood through the triple bottom line framework. SP requires balanced development across economic, environmental, and social dimensions^7,74^. For instance, in the environmental dimension, resilient firms are more likely to maintain their investments in resource conservation and pollution prevention during crises, avoiding the sacrifice of environmental commitments due to short-term pressures^44^. In the economic and social dimensions, resilience helps stabilize operations, safeguard employee well-being, and sustain community engagement, thereby supporting the achievement of SP^54,75^. Based on the above analysis, the following hypothesis is proposed:

##### H6

OR has a positive effect on SP.

This study does not hypothesize a direct effect of OA on SP. OA embodies a dynamic capability focused on short-term responsiveness, emphasizing the rapid sensing of market changes and resource reconfiguration^66^. In contrast, SP represents a strategic outcome formed over the long term from the integration of the economic, environmental, and social triple bottom lines, relying on sustained commitment to responsibility, stable stakeholder relationships, and shock-resistant OR. Agility itself is value-neutral; its sustainable value needs to be realized through translation into GI practices or the formation of OR. Therefore, this paper regards OA as an upstream capability that indirectly drives SP through GI and OR, rather than a direct antecedent.

#### Green innovation, organizational resilience, and sustainable performance

GI provides crucial support for building OR by expanding the firm’s technological capability frontier and market response space. Cleaner production, energy-saving processes, recycling, and green supply chain collaboration reduce resource dependency intensity and pollution risks, increase the substitutability of input factors, and mitigate the firm’s vulnerability in the face of supply disruptions, price fluctuations, and tightening regulations. The technological diversification and process optimization brought about by GI in this process enhance the firm’s resource substitutability and functional redundancy when confronting environmental shocks^76,77^. From a social perspective, the environmental legitimacy and stakeholder trust accumulated through GI constitute key social capital for firms to maintain operations and secure external support during crises^43^. Therefore, GI not only reduces the inherent environmental vulnerability of the firm but also systematically enhances its capacity for adaptation, recovery, and regeneration under disruption by reconfiguring its resource base and relational networks. Accordingly, the following hypothesis is proposed:

##### H7

GI has a positive effect on OR.

The NRBV regards GI as a mechanism through which firms transform environmental pressures into competitive advantage, linking it to the economic, environmental, and social dimensions of SP via the three pathways of pollution prevention, product stewardship, and sustainable development^7,25,36^. Firstly, through pollution prevention, resource efficiency improvements, and the application of clean technologies, GI significantly reduces energy consumption, emissions, and waste levels, thereby enhancing environmental performance^25,61^. Green product and green process innovations often lead to cost savings, quality improvements, and differentiated positioning, improving financial returns and market competitiveness^78,79^. GI is also linked to corporate social performance. By improving compliance and responding to community and customer demands for responsible production, it enhances organizational legitimacy and stakeholder satisfaction, thereby advancing the firm’s sustainable provision capabilities in the social and economic dimensions^80,81^. From the NRBV perspective, this signifies that firms translate their environmentally oriented capability accumulation into a competitive advantage for SP through GI. Accordingly, the following hypothesis is proposed:

##### H8

GI has a positive effect on SP.

#### Mediating roles of green innovation, organizational agility, and organizational resilience

The preceding hypotheses posit a direct effect of AIA on SP, as well as direct relationships between AIA and key mediating variables such as OA, GI, and OR. However, the mechanism through which AIA influences SP is far more complex than a single direct path. According to DCT, technological resources must undergo the evolutionary organizational process of “sensing-seizing-reconfiguring” to be translated into sustained competitive advantage^29^. Simultaneously, the NRBV posits that environmentally oriented technological practices are a key mechanism for firms to transform ecological constraints into sustainable value^25,36^. The integration of these two perspectives reveals the underlying logic of how AIA affects SP: AIA, as a higher-order technological resource and process, first enables and reshapes organizational dynamic capabilities (OA, OR) and green technological practices (GI). These capabilities and practices then, through synergistic and sequential transmission, collectively drive the firm’s SP across economic, environmental, and social dimensions. Therefore, this study proposes a set of mediation hypotheses to uncover these complex indirect pathways.

##### H9a

GI mediates the relationship between AIA and SP.

##### H9b

GI and OR sequentially mediate the relationship between AIA and SP.

##### H9c

OA and GI sequentially mediate the relationship between AIA and SP.

##### H9d

OA and OR sequentially mediate the relationship between AIA and SP.

##### H9e

OA, GI, and OR sequentially mediate the relationship between AIA and SP in a multi-stage chain.

## Methodology

### MASEM method

This study employs MASEM to examine the mechanisms linking AIA and SP. This methodological choice is driven by the inherent requirements of the research question: it necessitates not only identifying the direct effect between the two constructs but also simultaneously estimating the complete pathways through which AIA influences SP via multiple mediators within an integrated framework. Mediation analysis explores relationships established between independent and dependent variables through intervening variables^23^. Traditional meta-analysis typically synthesizes evidence for individual bivariate relationships, making it difficult to concurrently estimate and compare complete mediation pathways within a single framework. However, MASEM integrates evidence from multiple independent studies into a unified theoretical model, enabling the simultaneous testing of the overall pathways. This alignment between method and the complex structure of the theoretical problem enhances analytical rigor^27^.

The evidence-synthesizing nature of MASEM also provides a methodological foundation for the credibility and generalizability of the findings. First, the evidence is drawn from multiple independent studies involving diverse subjects and contexts. Robust patterns emerging across studies reduce the impact of contingency within any single context. Second, the research process emphasizes auditability. Adherence to systematic review and meta-analysis reporting guidelines in literature search, screening, and coding ensures a transparent process, serving as a procedural safeguard for credibility^82^. Third, this study employs a random-effects model for estimation. This model accommodates genuine heterogeneity in effects across studies, with the overall estimate reflecting the average relationship across the population of studies, making it more suitable for generalizing common trends across comparable contexts^83,84^. Consequently, by utilizing MASEM, this study elucidates both the direct effect and the multiple mediation pathways between AIA and SP, offering an empirical reference for understanding the mechanisms through which AIA influences SP in broader contexts.

### Data

A systematic literature search was conducted following the PRISMA guidelines^82^. The search timeframe was set from 2000 to 2025. In determining the search keywords, we referenced existing research and scholarly recommendations in related fields, ultimately summarizing five core categories of terms. The first category comprises AI-related keywords, including “artificial intelligence” and “artificial intelligence assimilation”^6,13^. The second category consists of green innovation-related terms, encompassing “green innovation”, “eco-innovation”, and “environmental innovation”^85^. The third category includes organizational agility-related terminology, namely “organizational agility”, “enterprise agility”, “firm agility”, and “corporate agility”^69,86–88^. The fourth category contains organizational resilience keywords: “organizational resilience”, “enterprise resilience”, “firm resilience”, and “corporate resilience”^89,90^. The fifth category involves sustainable performance-related vocabulary, including “sustainable performance” and “sustainability performance”^91,92^.

This study utilized Web of Science (WoS) and Scopus as the core databases, supplemented by Google Scholar, to ensure the comprehensiveness and representativeness of the retrieved literature. Prior research indicates that combining WoS, Scopus, and Google Scholar effectively balances search precision and recall^93^. These three databases perform well in terms of breadth of literature coverage and continuous updating. Adopting this comprehensive search strategy helps mitigate literature omissions caused by incomplete coverage of any single database or publication bias^94,95^, thereby providing a reliable data foundation for subsequent analysis.

**Fig. 2:**
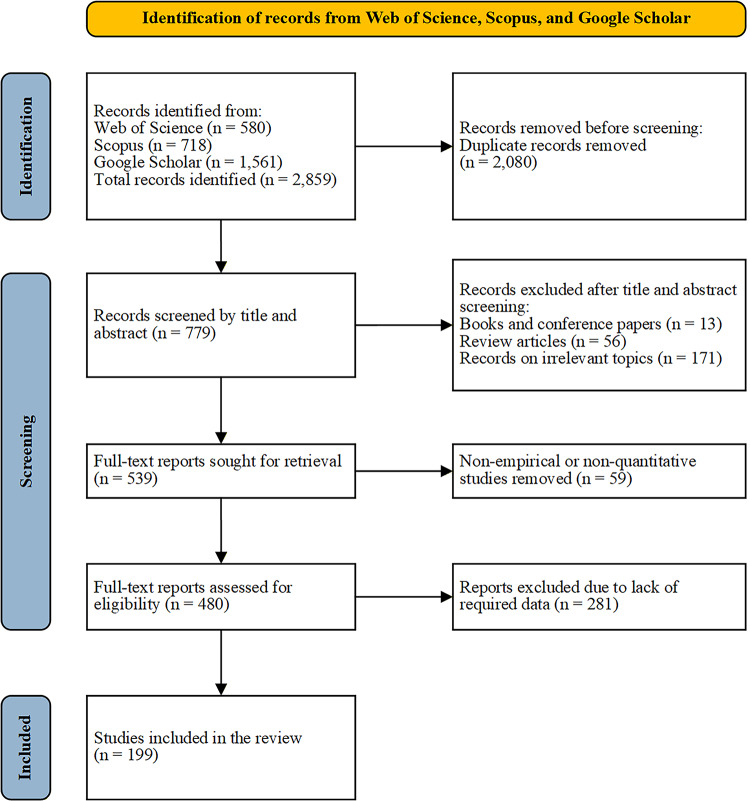
Study selection flow diagram.

The study selection process is illustrated in Fig. 2. Through the systematic literature search, we initially identified a total of 2,859 records, comprising 580 from WoS, 718 from Scopus, and 1,561 from Google Scholar. After removing 2,080 duplicate records, 779 unique records remained for the screening phase. In the initial screening, we evaluated the titles and abstracts of these 779 records and excluded 240 records. The primary reasons for exclusion were: non-journal publications (e.g., books and conference proceedings, *n* = 13), review articles (*n* = 56), and studies unrelated to the research topic (*n* = 171). Subsequently, we retrieved the full texts of the remaining 539 articles and conducted a detailed assessment of their eligibility. We further excluded 59 studies that were non-empirical or non-quantitative, as they could not provide the necessary data for analysis. Furthermore, 281 articles were excluded because they did not report key statistical information (such as correlation coefficients or other convertible effect size metrics) or did not report sample sizes. Ultimately, 199 studies met all the inclusion criteria and were retained for subsequent analysis. Generally, a minimum of two studies can suffice for conducting a meta-analysis^96^. The final inclusion of 199 studies in this research significantly exceeds this minimum threshold, thus providing a sufficient sample size suitable for subsequent analyses.

### Coding

To establish the data foundation for the MASEM, this study required the construction of a complete correlation matrix encompassing all core variables (AI assimilation, green innovation, organizational agility, organizational resilience, and sustainable performance). As the specific variable names used in individual primary studies might not be consistent, we uniformly identified them based on their conceptual definitions. AIA refers to the process by which firms continuously embed AI technologies into business processes, organizational systems, and daily operations, transforming them into repeatable organizational capabilities^13^. SP denotes a firm’s comprehensive performance across economic, environmental, and social dimensions^7^. GI refers to the introduction of environmentally oriented innovation activities into products, processes, or management^61,85^. OA indicates a firm’s ability to rapidly sense environmental changes and flexibly adjust resource allocation, encompassing aspects such as seizing market opportunities and operational adjustment flexibility^66,69^. OR signifies a firm’s capacity to maintain functionality, achieve recovery, and evolve continuously in the face of disruptions, including risk anticipation, crisis response, and adaptive learning^39,67^.

Pearson correlation coefficients reported in the primary studies were extracted as the basis for the effect size metric^27,97^. The data extraction was independently performed by two researchers to ensure objectivity and minimize bias. Any discrepancies arising during this process were resolved through discussion and consensus, following the PRISMA guidelines. Given that research at the intersection of AI and SP is still in a nascent stage, and to obtain a sufficient number of studies, approximate correlation coefficients were calculated using the conversion formula proposed by Peterson and Brown^98^ for studies that only provided standardized regression coefficients. For theoretical constructs measured via multiple dimensions, composite correlation coefficients were computed based on the intercorrelations among the sub-dimensions, following the method suggested by Schmidt and Hunter^99^.

In the effect size integration phase, heterogeneity tests were first conducted on the relationships between variables to objectively determine the appropriate integration model. If the heterogeneity test result was not significant, a fixed-effects model was employed for integration; otherwise, a random-effects model was used, acknowledging true variation between studies and making the results more generalizable^83,100^. All correlation coefficients were first transformed using Fisher’s *z* transformation to improve distributional properties. They were then weighted by the inverse of their variance and averaged on the *z* scale. The synthesized results were subsequently back-transformed to the correlation coefficient metric, forming the pooled correlation matrix used for the structural equation modeling^27,101,102^. To implement MASEM, we extracted the reliability coefficients (Cronbach’s *α* or Composite Reliability) for the variables from the included studies, calculated their cross-study averages, and used these to correct for measurement error^103,104^. As the primary studies had varying sample sizes, the harmonic mean was adopted as the sample size for the SEM analysis^105^. Finally, Amos software was utilized for the SEM tests.

## Results

### Bivariate heterogeneity and correlation tests

Following the methodology described above, tests for bivariate heterogeneity, correlations, and publication bias were conducted. Table 1 summarizes the integrated bivariate correlation results for the ten core relationships in this study.

Heterogeneity tests revealed that the *Q*-statistics for all relationships were highly significant, exceeding their respective degrees of freedom. Concurrently, the *I*^2^ statistics were high (ranging from 88.460% to 99.671%). This clearly indicates substantial and true heterogeneity in effect sizes across the primary studies. The random-effects model, which acknowledges and accounts for this true variation between studies, yields more general estimates than the fixed-effects model. Therefore, this study employed and reports the adjusted weighted correlation coefficient *r* under the random-effects model for all bivariate relationships and conducted significance tests on them.

The results show that the weighted mean correlation coefficients for all relationships were statistically significant (*p* < 0.05). The effect sizes for these relationships ranged from 0.395 (OA-SP) to 0.612 (OA-OR). According to the guidelines provided by Cohen^106^, the vast majority of relationships (9 out of 10) in this study reached or exceeded the threshold for medium-to-strong correlations. It is noteworthy that the confidence intervals were wider for some relationships, primarily those with a smaller number of available studies (e.g., GI-OR: *r* = 0.575; OR-SP: *r* = 0.501). The common characteristic of these two relationships is their relatively smaller number of study samples. Even for these relationships with the most limited evidence, the direction remained positive and the null hypothesis of zero correlation was rejected at conventional significance levels. Therefore, while maintaining caution, these paths were retained and included in the overall model^103^.

**Table 1:** Analysis of bivariate correlation.

Relationships	K	Fail-safe *N*	*N*	Weighted average correlation		Confidence interval (95%)		Z	*p*	Q	I^2^ (%)
				*r*	SE	Lower limit	Upper limit				
AIA-GI	33	32,003	41,303	0.461	0.050	0.373	0.540	9.226	0.000	3,064.229	98.956
AIA-OA	18	6,598	5,147	0.496	0.047	0.417	0.568	10.591	0.000	221.568	92.327
AIA-OR	11	2,401	2,828	0.500	0.104	0.315	0.649	4.809	0.000	363.088	97.246
AIA-SP	66	4,076	8,488	0.451	0.031	0.397	0.502	14.449	0.000	4,857.970	98.662
GI-OA	5	412	1,878	0.400	0.065	0.281	0.507	6.169	0.000	34.663	88.460
GI-OR	4	481	27,284	0.575	0.293	0.001	0.864	1.962	0.049	643.134	99.534
GI-SP	98	243,246	31,243	0.503	0.031	0.451	0.551	16.111	0.000	4,467.487	97.829
OA-OR	8	2,403	2,988	0.612	0.074	0.496	0.706	8.315	0.000	129.640	94.600
OA-SP	17	3,640	5,931	0.395	0.067	0.273	0.505	5.922	0.000	455.686	96.489
OR-SP	15	2,962	19,936	0.501	0.170	0.183	0.724	2.950	0.003	4,250.867	99.671

Furthermore, the Fail-safe *N* values were all substantially higher than the recommended threshold of 5*K* + 10 (where *K* is the number of studies). This suggests that an implausibly large number of unpublished null results would be required to overturn the current significant conclusions, a scenario unlikely to occur in reality. Thus, it is reasonable to conclude that the sample data in this study are unlikely to be substantially affected by publication bias^107^. This evidence provides a foundation for the subsequent hypothesis testing and path analysis.

### SEM model results

**Fig. 3:**
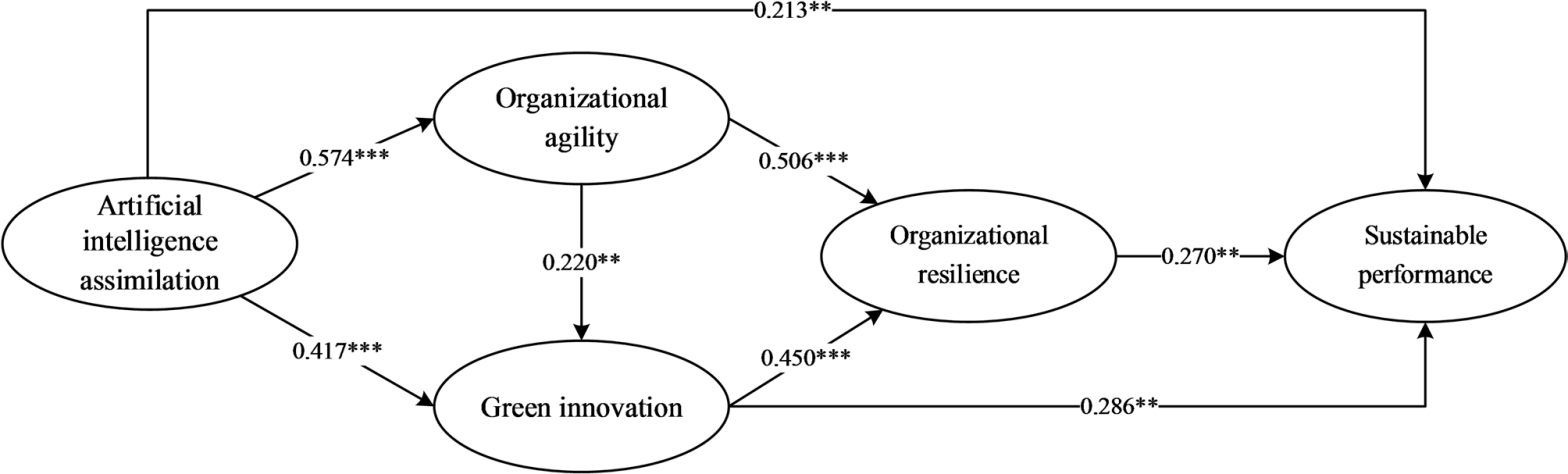
Results of the SEM direct effects. *Note*: Significance: ***p < 0.001; **p < 0.01.

In the structural equation modeling stage, this study first calculated the weighted correlation coefficients between variables and the reliabilities of each variable based on the meta-analytic results (see Table 2), using a harmonic mean of 262 as the sample size for model estimation. Figure 3 presents the results of the SEM direct effects. The model fit indices show: The chi-square test result of χ² = 2.177, *p* = 0.337, indicating no significant difference between the correlation matrix derived from the theoretical model and the observed correlation matrix obtained from the meta-analysis; therefore, the model was not rejected^108^. Further referencing the fit criteria by Hu and Bentler^109^, the SRMR was 0.012 (< 0.08), TLI was 0.998 (> 0.95), and RMSEA was 0.018 (< 0.06), all reaching excellent levels. Additionally, the GFI and AGFI were 0.997 and 0.975, respectively, both exceeding the recommended benchmark of 0.90^110^. Judging by the comprehensive set of indices, the theoretical model demonstrates a good overall fit.

**Table 2:** Variable correlation matrix.

	AIA	GI	OA	OR	SP
AIA	0.865				
GI	0.461	0.855			
OA	0.496	0.400	0.885		
OR	0.500	0.575	0.612	0.844	
SP	0.451	0.503	0.395	0.501	0.869

*Note:* The lower triangle of the matrix presents the adjusted weighted correlation coefficients between variables, and the diagonal presents the average reliability of each variable.

The results of the direct effect tests are presented in Table 3. Specifically, AIA had significant positive effects on OA (*β* = 0.574, *p* < 0.001), GI (*β* = 0.417, *p* < 0.001), and SP (*β* = 0.213, *p* < 0.01). Concurrently, OA drove OR (*β* = 0.506, *p* < 0.001) and also significantly promoted GI (*β* = 0.220, *p* < 0.01). Furthermore, GI effectively enhanced both OR (*β* = 0.450, *p* < 0.001) and SP (*β* = 0.286, *p* < 0.01). Finally, OR also exerted a positive influence on SP (*β* = 0.270, *p* < 0.01). In summary, Hypotheses H1 through H8 were all supported.

**Table 3:** Direct effect test results.

Hypothesis	Path	Unstandardized coefficient	Standardized coefficient	SE	CR	*p*	Results
H1	AIA→OA	0.575***	0.574***	0.061	9.341	0.000	Supported
H2	AIA→GI	0.417***	0.417***	0.080	5.191	0.000	Supported
H3	AIA→SP	0.213**	0.213**	0.075	2.827	0.005	Supported
H4	OA→OR	0.506***	0.506***	0.059	8.615	0.000	Supported
H5	OA→GI	0.220**	0.220**	0.079	2.775	0.006	Supported
H6	OR→SP	0.270**	0.270**	0.092	2.939	0.003	Supported
H7	GI→OR	0.450***	0.450***	0.060	7.536	0.000	Supported
H8	GI→SP	0.286**	0.286**	0.092	3.099	0.002	Supported

Notes: ***p < 0.001; **p < 0.01; *p < 0.05. AIA: AI assimilation; GI: Green innovation; OA: Organizational agility; OR: Organizational resilience; SP: Sustainable performance.

The total, direct, and indirect effects were decomposed and tested using the Bootstrap method (5000 resamples, 95% bias-corrected confidence intervals). The results (see Table 4) show that the 95% bias-corrected confidence intervals for all paths did not include zero. The total effect of AIA on SP was 0.513. Effect decomposition revealed a direct effect of 0.213, accounting for 41.52% of the total effect; the combined indirect effects were 0.300, accounting for 58.48% of the total effect. The dominance of the indirect effects indicates that the impact of AIA on SP is primarily realized through the mediating roles of organizational capabilities and innovation mechanisms. Specifically, all five indirect paths (H9a-e) were significant. Among them, the simple mediation path through GI was the strongest (effect = 0.119), accounting for 23.20% of the total effect, indicating that green innovation is the primary mechanism through which AIA promotes SP (H9a supported). Next, the path from AIA through OA and OR to SP had an effect of 0.078, accounting for 15.20% of the total effect (H9d supported). The path from AIA through GI and OR to SP had an effect of 0.051, accounting for 9.94%, indicating that green innovation not only directly promotes performance but also exerts an indirect effect by enhancing OR (H9b supported). Furthermore, the paths from AIA through OA and GI to SP and from AIA through OA, GI, and OR to SP accounted for 7.02% and 2.92% of the total effect, respectively, verifying the facilitating role of agility on green innovation and the complete quadruple serial mediation chain (H9c and H9e supported). Collectively, these results support the proposed multiple mediation hypotheses (H9a-e), revealing the complex network of mechanisms through which AIA influences SP.

**Table 4:** Mediation test analysis.

	Path	Effect size	SE	Bias-corrected 95% CI		*p*	Share of total effect
				Lower Limit	Upper Limit		
Total effect		0.513	0.066	0.386	0.647	0.000	100.00%
Direct effect	AIA→SP	0.213	0.078	0.061	0.365	0.006	41.52%
Indirect effect	AIA→GI→SP	0.119	0.046	0.043	0.226	0.001	23.20%
	AIA→OA→OR→SP	0.078	0.031	0.024	0.144	0.004	15.20%
	AIA→GI→OR→SP	0.051	0.023	0.016	0.110	0.003	9.94%
	AIA→OA→GI→SP	0.036	0.019	0.009	0.087	0.004	7.02%
	AIA→OA→GI→OR→SP	0.015	0.008	0.004	0.040	0.004	2.92%

*Notes:* Bootstrap samples = 5,000. AIA: AI assimilation; GI: Green innovation; OA: Organizational agility; OR: Organizational resilience; SP: Sustainable performance.

## Discussion

### The AIA–SP nexus within dynamic capabilities

Grounded in DCT and the NRBV, this study systematically examined the mechanism through which AIA affects SP using MASEM. The results indicate that AIA has a significant positive effect on SP. This finding aligns with the conclusions of Mikalef and Gupta^2^, and Wamba-Taguimdje et al.^4^, confirming the strategic value of AI technology in corporate sustainable development.

Our conclusion addresses the divergent findings regarding AI’s performance outcomes. From the perspective of DCT, this divergence may stem from deficiencies in the process of transforming AI resources into organizational capabilities. This study found that the direct effect of AIA on SP accounts for only 41.52% of the total effect, whereas the indirect effects account for 58.48%. This suggests that the “crucial leap” from AI resources to SP primarily relies on the intermediate transformation through organizational capabilities, rather than on mere technological deployment.

The findings further reveal that GI plays a pivotal bridging role between AIA and SP. This discovery corroborates the NRBV perspective that environmentally oriented technological practices are a key mechanism for firms to transform ecological constraints into competitive advantage^25,62^. This conclusion is also supported by recent views suggesting that AI significantly enhances a firm’s GI capability by improving resource allocation efficiency, optimizing energy use, and reducing pollution emissions^8,64^, thereby promoting SP. This indicates that if firms position AI solely as a tool for operational efficiency, neglecting its strategic value in GI, they will struggle to fully realize its potential for enabling SP.

OA plays a dual role in the process through which AIA influences SP. First, OA is a direct outcome of AIA. AI technologies, through real-time data processing, pattern recognition, and intelligent decision support, significantly enhance a firm’s speed in capturing market signals and its flexibility in responding to environmental changes. Research by Khan and Kwan^111^ confirms that AI significantly promotes OA, which in turn improves environmental performance. The study by Chatterjee et al.^60^ also indicates that embedding AI into business processes enhances a firm’s speed and flexibility in identifying and responding to market signals. Second, OA acts as a bridge in two critical pathways. By accelerating resource recombination and knowledge integration, OA provides the necessary organizational conditions for GI. Rabal-Conesa et al.^72^ found that OA significantly promotes GI outcomes by enhancing cross-departmental coordination and knowledge recombination capabilities, which is consistent with the conclusion of this study.

Simultaneously, OA, through continuous daily responsive practices, gradually sediments into OR. While agility focuses on short-term, high-frequency responsiveness, the repeated application of this capability leads to the accumulation of experience and the development of preparedness mechanisms for non-routine situations within the organization. A similar view posits that dynamic capabilities often influence performance by enhancing resilience, and the rapid adaptation capacity supported by agility translates into more stable capabilities for withstanding pressure and recovering^68^. OA thus serves as a key nexus connecting short-term responsiveness with long-term adaptive capacity.

OR plays a long-term safeguarding role in the process through which AIA influences SP. Unlike the short-term, routine, and high-frequency characteristics of OA, OR emphasizes a firm’s ability to maintain functional stability, achieve effective recovery, and learn and evolve when facing major disruptions^67,90^. In other words, agility concerns rapid responses to handle daily fluctuations, whereas resilience focuses on stable endurance to cope with systemic shocks. This distinction holds significant conceptual and practical importance. Research by Yan et al.^44^ supports this assertion, finding a mutually reinforcing relationship between OR and sustainability, with operational redundancy playing a key role. The findings further reveal that, in the context of digital transformation, this OR does not emerge spontaneously but is cultivated gradually through the accumulation of agility driven by AIA and the practices of GI.

The study confirms the existence of the serial mediation paths involving OA, GI, and OR between AIA and SP. Agility enables firms to respond rapidly and initiate GI. The continuous implementation of GI gradually fosters resilient mechanisms for coping with environmental pressures. Resilience, in turn, ensures that firms maintain their SP advantage over the long term. The realization of SP cannot rely solely on AI technology investment or GI project implementation; it requires the systematic cultivation and integration of multiple dynamic capabilities. This aligns closely with the strategic resource integration logic emphasized by the NRBV^25,36,62^. This finding resonates with existing research on the dynamic, mutually constitutive relationship between agility and resilience^112^ and underscores the strategic value of their synergy in the context of increasing pressures for sustainable transformation.

### Implications for theory

First, this study synthesizes evidence to support a stable positive association between AIA and SP and elucidates the primary mechanisms underlying this relationship from a dynamic capabilities perspective. Extant literature presents divergent conclusions regarding the AIA-SP link, largely attributable to variations in sample sources, variable operationalizations, and model specifications. By integrating evidence from multiple empirical sources, this study arrives at a more consistent judgment at the aggregate level, concluding that AIA exerts a positive and robust influence on SP. This influence is primarily realized through the evolution and interaction of a set of dynamic capabilities, namely OA, GI, and OR. This explanation reframes the connection between technological input and performance output into a process of capability building and evolution, thereby providing a middle-range theoretical explanation for how AI technology creates sustainable value.

Second, from a dynamic capabilities perspective, this study distinguishes between OA and OR and posits a sequential relationship between them. Some prior research subsumes agility within the broader construct of resilience, emphasizing the importance of rapid sensing, flexible adjustment, and continuous adaptation for resilience development^39,113^. Other studies discuss agility and resilience separately but lack systematic examination of how they are interrelated at the organizational level to form an evolutionary capability chain^33,114,115^. This study posits that agility is oriented toward rapid responsiveness, whereas resilience is oriented toward long-term adaptation and continuity. The results indicate that agility is more likely to influence outcomes by promoting GI, which in turn fosters the accumulation of resilience, ultimately linking to SP. This pathway reveals the synergistic order between rapid response capabilities and enduring adaptation capabilities within organizations pursuing sustainability objectives.

Third, this study embeds strategic value orientation into the capability evolution process, thereby enhancing the applicability of the NRBV and DCT for explaining SP in the context of digital transformation. On one hand, AI as a strategic digital resource investment, through its assimilation process, activates GI initiatives such as environmental protection and pollution prevention, which are subsequently translated into SP. On the other hand, by incorporating OA and OR into the capability evolution path of sensing, seizing, and reconfiguring, this study clarifies their roles as the dynamic capabilities that transform technological resources into SP, consequently enriching the theoretical details of DCT regarding the transmission mechanisms leading to SP. This integrated framework demonstrates that AIA propels the transformation of organizational sustainability strategy into dynamic capabilities and fosters synergistic mechanisms within the organization, thus providing a theoretical basis for the formation of sustainable competitive advantage.

### Implications for practice

The findings of this study offer the following implications for how organizations can effectively advance sustainable development during their digital and intelligent transformation:

First, organizations need to formulate an institutionalized system for transforming technology adoption into capability development. The value realization of technologies like AI depends on a managed process that embeds them within the organization and sequentially activates agility, GI, and resilience. Consequently, the managerial focus should shift from evaluating the technology itself to designing and governing this transformation process. This requires linking technology investment decisions directly to explicit goals for sustainable capability development, ensuring that resource allocation effectively drives the directional evolution of internal organizational capabilities.

Second, organizations should design management mechanisms that coordinate exploratory front-end activities with consolidating back-end functions. This study distinguishes between the rapid response function of agility and the long-term stabilizing function of resilience. This distinction necessitates differentiated managerial arrangements. Front-end units should be empowered to use technology for rapidly validating sustainable solutions, with emphasis placed on their learning velocity and knowledge generation. Concurrently, back-end functions should be established to integrate validated knowledge into the organization’s standard procedures, redundant resources, and risk contingency plans, focusing on their institutionalization effectiveness. The integration of these two aspects requires clear definitions of authority, responsibility, processes, and resource allocation.

Finally, organizations are required to internalize sustainability as a fundamental criterion for technology assessment and capability development. Technology-driven capability evolution requires a clear value anchor. Organizations need to establish sustainability screening criteria during the technology selection phase, incorporate environmental and social objectives into capability-building initiatives, and include corresponding sustainable outputs in performance evaluations. By translating the concept of sustainability into concrete decision-making bases and evaluation metrics, organizations can steer the development trajectory of their capabilities, synchronizing this growth with the accumulation of long-term legitimacy.

## Conclusion

Grounded in the NRBV and DCT, this study employed MASEM to systematically investigate the mechanism through which AIA affects corporate SP. The main conclusions are as follows: First, AIA directly promotes SP and also exerts indirect effects through the multiple mediating pathways of OA, GI, and OR. Second, GI is the core pathway through which AIA influences SP, highlighting the strategic value of GI practices in connecting AI technology with sustainable development goals. Third, OA and OR are confirmed as two interrelated yet functionally complementary capability dimensions, together constituting the organizational capability foundation for AI-enabled sustainable development. Fourth, this research validates the complex mechanism of how AIA affects SP, indicating that firms need to establish a synergistic evolution system of “technology-capability-innovation-performance” to realize the sustainable development value of AI.

The findings of this study have been validated through empirical data from multiple diverse organizational and industrial contexts, thereby demonstrating strong generalizability. However, due to the complexity of specific contexts, these conclusions may vary across different types of organizations and national settings, particularly under varying institutional, cultural, and digital transformation stages. Owing to the limitations of existing literature data, this study did not delve into the moderating effects of these contextual factors on the results. Therefore, future research could further explore the impact mechanisms of AIA on SP across different industries, countries, and organizational structures. Additionally, employing longitudinal research designs to deepen the identification of causal relationships would contribute to a more comprehensive understanding of the effects of AIA on performance.

## Data Availability

The data supporting the conclusions of this article are available from the corresponding author upon reasonable request.

## Abbreviations

AI: Artificial intelligence
AIA: Artificial intelligence assimilation
GI: Green innovation
OA: Organizational agility
OR: Organizational resilience
SP: Sustainable performance

## **Code availability**

No custom code, scripts, or algorithms were used in this study. All analyses were conducted using Comprehensive Meta-Analysis (CMA) and IBM SPSS Amos software. Therefore, no code is available.
